# A Newly Developed Diabetes Risk Index, Based on Lipoprotein Subfractions and Branched Chain Amino Acids, is Associated with Incident Type 2 Diabetes Mellitus in the PREVEND Cohort

**DOI:** 10.3390/jcm9092781

**Published:** 2020-08-27

**Authors:** Jose L. Flores-Guerrero, Eke. G. Gruppen, Margery A. Connelly, Irina Shalaurova, James D. Otvos, Erwin Garcia, Stephan J. L. Bakker, Robin P. F. Dullaart

**Affiliations:** 1 Division of Nephrology, Department of Internal Medicine, University of Groningen, University Medical Center Groningen, 9713 GZ Groningen, The Netherlands; e.g.gruppen@umcg.nl (E.G.G.); s.j.l.bakker@umcg.nl (S.J.L.B.); 2 Laboratory Corporation of America Holdings (LabCorp), Morrisville, NC 27560, USA; connem5@labcorp.com (M.A.C.); shalaui@labcorp.com (I.S.); otvosj@labcorp.com (J.D.O.); garce14@labcorp.com (E.G.); 3 Division of Endocrinology, Department of Internal Medicine, University of Groningen, University Medical Center Groningen, 9713 GZ Groningen, The Netherlands; dull.fam@12move.nl

**Keywords:** DRI, insulin resistance, LP-IR, branched chain amino acids, type 2 diabetes, biomarkers

## Abstract

Objective: Evaluate the ability of a newly developed diabetes risk score, the Diabetes Risk Index (DRI), to predict incident type 2 diabetes mellitus (T2D) in a large adult population. Methods: The DRI was developed by combining the Lipoprotein Insulin Resistance Index (LP-IR), calculated from 6 lipoprotein subspecies and size parameters, and the branched chain amino acids, valine and leucine, all of which have been shown previously to be associated with future T2D. DRI scores were calculated in a total of 6134 nondiabetic men and women in the Prevention of Renal and Vascular End-Stage Disease (PREVEND) Study. Cox proportional hazards regression was used to evaluate the association of DRI scores with incident T2D. Results: During a median follow-up of 8.5 years, 306 new T2D cases were ascertained. In analyses adjusted for age and sex, there was a significant association between DRI scores and incident T2D with the hazard ratio (HR) for the highest versus lowest quartile being 12.07 (95% confidence interval: 6.97–20.89, *p* < 0.001). After additional adjustment for body mass index (BMI), family history of T2D, alcohol consumption, diastolic blood pressure, total cholesterol, triglycerides, HDL cholesterol and HOMA-IR, the HR was attenuated but remained significant (HR 3.20 (1.73–5.95), *p* = 0.001). Similar results were obtained when DRI was analyzed as HR per 1 SD increase (HR 1.37 (1.14–1.65), *p* < 0.001). The Kaplan–Meier plot demonstrated that patients in the highest quartile of DRI scores presented at higher risk (*p*-value for log-rank test <0.001). Conclusions: Higher DRI scores are associated with an increased risk of T2D. The association is independent of clinical risk factors for T2D including HOMA-IR, BMI and conventional lipids.

## 1. Introduction

In order to curtail the growing epidemic of obesity and type 2 diabetes mellitus (T2D), new clinical practice guidelines recommend structured lifestyle modification and/or pharmacological intervention for patients who are at high risk of developing T2D [1,2]. Over 80 million adults in the United States alone qualify as being high risk based on their glycemic status [3]. Early intervention in individuals who are insulin resistant but have not yet shown signs of impaired glucose tolerance or fasting dysglycemia, may prevent or delay the progression to T2D [4,5]. Multiple clinical studies have also shown that lifestyle interventions and pharmacological therapies are able to delay to an important extent the onset of T2D even in subjects who are already experiencing dysglycemia [6,7,8,9]. Genome-wide association studies have identified more than 200 genetic loci which are associated with development of T2D [10,11]. Currently, application of this genetic information is more likely to be used in support of studies on pathophysiological understanding of T2D rather than to be applied in actual clinical risk prediction, where biomarkers still are most promising [12]. However, disappointingly, few biomarker candidates provide improvement of T2D risk prediction over conventional measures of glycaemia and adiposity [12]. Therefore, there remains a clinical need for diagnostic tools that identify high risk patients in order to employ therapeutic measures early in the course of worsening dysglycemia.

The Lipoprotein Insulin Resistance Index (LP-IR) is a multimarker score derived from six lipoprotein subclass and size parameters measured clinically by the high-throughput *NMR LipoProfile*^®^ test [13,14]. LP-IR scores (0–100) reflect the magnitude of insulin resistance in individual patients and exhibit strong associations with homeostatic model assessment of insulin resistance (HOMA-IR) and the glucose disposal rate (GDR) assessed by hyperinsulinemic-euglycemic clamp, and have been shown to reflect both peripheral and hepatic insulin resistance [15].

LP-IR scores have been shown recently to predict future T2D in several studies, namely the Multi-Ethnic Study of Atherosclerosis (MESA) [16], Women’s Health Study [17], Justification for the Use of Statins in Prevention: an Intervention Trial Evaluating Rosuvastatin trial [18] and Prevention of Renal and Vascular End Stage Disease (PREVEND) [19]. In all four of these studies, LP-IR scores were strongly associated with incident T2D even after adjustment for known T2D risk factors and in individuals at low risk for T2D based on their clinical profiles [16,17,18,19]. Importantly, lifestyle interventions have been shown to lower LP-IR scores, suggesting that LP-IR may be useful for monitoring treatments that may prevent or delay the onset of T2D [20,21,22,23].

Plasma levels of branched chain amino acids (BCAA), i.e., leucine, valine, and isoleucine, have also been shown to be higher in insulin resistant conditions and to be associated with development of T2D [24,25,26,27,28,29]. Moreover, it has been proposed that reducing elevated BCAA levels may provide a therapeutic approach for treating insulin resistance [30]. Since concentrations of BCAA and other metabolites are measured at no incremental analytic cost during *NMR LipoProfile*^®^ testing [31], we hypothesized that a multimarker score combining LP-IR and BCAA would provide an enhanced clinical ability to stratify T2D risk in individuals with similar glucose levels. To this end, data from MESA [16] were used to develop a new multimarker called the Diabetes Risk Index (DRI).

The aim of this study was to assess the ability of DRI scores to predict future T2D in the PREVEND study, a large cohort of adults from the general population.

## 2. Materials and Methods

### 2.1. Study Design and Participants

The PREVEND study was approved by the local medical ethics committee at the University Medical Center Groningen (approval number: MEC96/01/022). All participants provided written informed consent and all procedures were conducted according to the Declaration of Helsinki. Details of the study design and recruitment have been described elsewhere [32]. Briefly, the PREVEND study is a Dutch cohort drawn from the general population of the city of Groningen in the northern part of the Netherlands. After exclusion of subjects with insulin-treated diabetes and pregnant women, all subjects with a urinary albumin concentration ≥10 mg/L were invited to participate (*n* = 7768), of whom 6000 accepted.

In addition, a random sample of 2592 individuals with a urinary albumin concentration <10 mg/L was included. These 8592 subjects (aged 28–75 years) completed the baseline survey (1997–1998). The second screening, which was the starting point of the current study, took place between 2001 and 2003 (*n* = 6892). For the current study, subjects with T2D at baseline, missing data on diabetes or glucose at baseline, and those with missing NMR or covariate data at baseline and follow-up were excluded, leaving 6134 subjects for the present analyses.

Follow-up time was defined as the period between the second screening round (baseline) and the date of ascertainment of T2D. Follow-up time was censored at 8.5 years. In case a person moved to an unknown destination, census date was the date of removal from the municipal registry. Incident cases of diabetes were ascertained if one or more of the following criteria were met: (1) fasting plasma glucose (FPG) ≥7.0 mmol/L (126 mg/dL); (2) random sample plasma glucose ≥11.1 mmol/L (200 mg/dL); (3) self-report of a physician diagnosis of T2D and (4) initiation of glucose-lowering medication use, retrieved from a central pharmacy registry [33,34].

### 2.2. Laboratory Measurements

Venous blood was obtained after an overnight fast. EDTA plasma samples were prepared by centrifugation at 4 °C as per manufacturer’s instructions. Total cholesterol (TC), high-density lipoprotein cholesterol (HDL-C) and triglycerides (TG) were measured on a Beckman Coulter^®^ AU680 Analyzer [35]. Fasting plasma glucose was measured by dry chemistry (Eastman Kodak, Rochester, NY, USA). HOMA-IR was calculated as fasting plasma insulin (µU/mL) × (FPG (mg/dL) × 0.055)/22.5 and values were log transformed for analysis.

EDTA plasma samples from the second screening were stored at <−70 °C until being shipped to LabCorp for *NMR LipoProfile*^®^ testing. NMR spectra were collected on a Vantera^®^ Clinical Analyzer and LP-IR scores were calculated and branched chain amino acids were quantified as previously described [13,14,31].

### 2.3. DRI Development

The DRI score was designed to improve upon the established performance of LP-IR as a clinical predictor of T2D by combining it with BCAA measures, elevated levels of which are linked to diabetes risk by possibly novel mechanisms [26,27]. To determine how best to combine LP-IR and BCAA to optimize T2D prediction, we employed logistic regression using the same dataset used for the development of LP-IR [15], namely baseline NMR data from MESA comprised of 4982 U.S. adults (mean age 62 years; range 45–84) without cardiovascular disease or diabetes, of whom 595 developed T2D during a mean 7.8-year follow-up [16]. Initial development work used total BCAA (sum of valine, leucine, and isoleucine), but subsequently it was found that better performance was achieved without isoleucine, possibly because measurement precision for isoleucine (ranging from 8.8–21.3% CV for within run and within lab imprecision) is not as good as that for valine (1.7–5.4% CV) and leucine (4.4–9.1% CV) [31]. In a logistic regression model including age, sex, race, and fasting glucose, both LP-IR and a BCAA parameter (valine + 2 × leucine) contributed independently to prediction of incident diabetes and the regression coefficients from this model were used as weighting factors for the following equation: DRI = 0.0167 (LP-IR) + 1.907 [ln (valine + 2 × leucine)]. For clinical use, the DRI values were transformed into a 1–100 score, using 1st and 99th percentile values to define the low and high limits of the range. The coefficients of variation for intra- and inter-assay precision ranged from 3.9–6.4% and 2.7–7.9% for LP-IR and DRI, respectively.

### 2.4. Statistical Analyses

All statistical analyses were performed with R language for statistical computing software [36], v. 3.6.2 and the integrated development environment (IDE) RStudio [37], v.1.2.5019.

For all analyses, two-sided *p* values <0.05 were considered statistically significant, except for interaction terms for which in agreement with existing literature, the level of significance was set at *p* < 0.10 [38].TG and HOMA-IR were log transformed when used as a continuous variable in the analysis.

Baseline characteristics were calculated across sex-specific quartiles of DRI scores. *p*-values across quartiles of DRI were determined by linear regression for continuous variables or chi-square test for categorical variables. Cox proportional hazards regression analysis was performed to examine the associations of DRI across quartiles calculated in the whole study population with the risk of developing T2D. In addition, hazards were calculated per 1 standard deviation (SD) increment of DRI. Hazard ratios (HR) were expressed with 95% confidence intervals (CI). Harrell’s c-index was calculated with and without the addition of DRI scores. To identify the best-fitting model, the Bayesian Information Criterion and the Akaike Information Criterion were computed [39,40]. In addition, the performance of the enriched model with LPIR and DRI in terms of true positive rates and false positive rate was evaluated with the McNemar test [41].

The Net Reclassification Index (NRI) [42] was calculated using the Framingham Offspring Study T2D risk score [43] with and without the addition of DRI scores as a continuous variable, considering predefined risk categories of type 2 diabetes development (<10%), intermediate (10% to 20%), and high (≥20%) [44].

## 3. Results

Of the PREVEND participants that completed the second round of screening, 6134 subjects who did not have T2D and had complete data available on DRI and covariates at the time of screening were included in this study. Baseline characteristics for these subjects, stratified by quartiles of DRI scores, can be found in Table 1. Participants with higher DRI scores were more likely to be men and were older in age. They were also more likely to have a parental history of T2D, be current smokers, consume more alcohol, and to be on lipid lowering or hypertensive medications. Those in the highest quartile of DRI scores had higher BMI, systolic and diastolic blood pressure, TC, TG, LDL-C, glucose, BCAA and LP-IR scores, and lower HDL-C. Baseline characteristics of sex-stratified quartiles of DRI show similar frequencies (Supplemental Table S1).

After a median (interquartile range) follow-up period of 8.5 (8.0–9.0) years, 306 new T2D cases were ascertained, 193 cases in men and 113 cases in women, 6.4% vs. 3.6%, respectively. Cox proportional hazards regression was used to evaluate the DRI scores with incident T2D (Table 2). The crude model revealed that DRI scores are associated with incident T2D with a hazard ratio (HR) for the highest quartile of 12.05 (95% confidence interval (CI): 7.12–20.41; *p* < 0.001). The association of DRI scores with future T2D remained significant even after adjustment for age, sex, BMI, family history of T2D, alcohol consumption, blood pressure, TC, TG, HDL-C and HOMA-IR. The HR for the highest quartile being 3.20 (1.73–5.95; *p* < 0.001). DRI per 1 standard deviation (SD) increment after full adjustment for clinical risk factors for T2D was 1.50 (1.25–1.79; *p* = 0.001).

**Figure 1:**
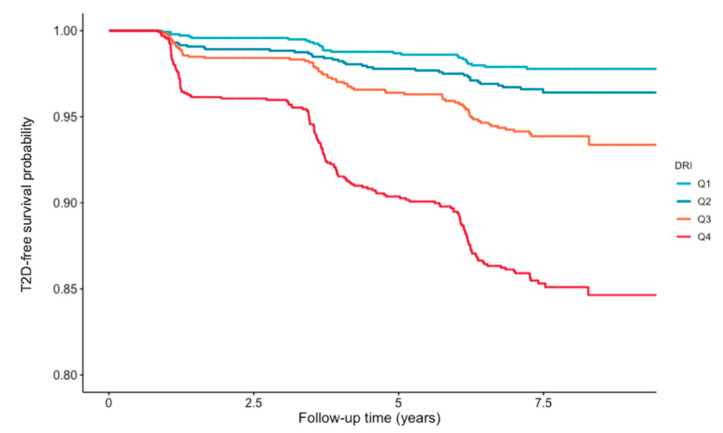
Kaplan–Meier survival curves for time to T2D diagnosis according to sex-stratified quartiles of DRI, by log-rank test (*p* < 0.001).

Similarly, Cox proportional hazard regression analysis was performed using sex-stratified quartiles of DRI scores. The crude model again revealed that DRI scores are associated with incident T2D with a hazard ratio (HR) for the highest quartile of 7.27 (95% CI: 4.84–10.92; *p* < 0.001). The association of DRI scores with future T2D remained significant even after adjusting for clinical T2D risk factors with a HR for the highest quartile being 1.80 (1.07–3.02; *p* < 0.001) (Table 3). The Kaplan–Meier curves for sex-stratified quartiles of DRI scores are shown in Figure 1. The data show that increasing quartiles of DRI scores corresponded to higher T2D incidence (log rank *p* < 0.001) (Figure 1).

To assess the performance of DRI, we calculated the Harrell’s C-index (95% CI) for the Framingham Offspring risk score (a traditional T2D risk assessment tool that takes into account age, sex, family history of T2D, BMI, blood pressure, TG, and glucose) to be 0.870 (0.869–0.870), which increased to 0.876 (0.875–0.877) after addition of DRI, a statistically significant improvement (*p* < 0.001). The Net Reclassification Index (NRI) was 0.41 (0.30–0.52; *p* < 0.001), denoting that when DRI was added to the model, more subjects were correctly re-classified than when the Framingham Offspring Study risk score was used alone.

The addition of LP-IR to the Framingham Offspring Study risk score allowed the proper reclassification of 27% of subjects who developed T2D, from a lower to a higher risk. Importantly, the addition of DRI allowed for 42% of the participants who developed T2D during the follow-up to be properly reclassified from a lower to a higher risk category.

Furthermore, when the LP-IR enriched model was compared against the DRI-enriched model, 24% of participants who developed T2D during the follow-up to be properly reclassified from a lower to a higher risk category, with a Net Reclassification Index (NRI) of 0.34 (0.22–0.45; *p* < 0.001).

The Framingham Offspring Study models enriched with LPIR and DRI were compared. The true positive rate for the DRI-enriched model was superior to the LPIR-enriched model (0.20 vs. 0.16, respectively, *p* < 0.05). Although the true negative rates of the DRI- and LPIR-enriched models were similar (0.95 vs. 0.95, *p* > 0.05), the difference with respect to the negative predictive values (0.0015) was statistically significance (*p* < 0.05), in favor to DRI-enriched model.

Based on the lowest Bayesian Information Criterion, the best-fit model for the T2D risk was the model enriched with DRI. The Bayesian Information Criterion for DRI-enriched model was 1863, while the LPIR-enriched model was 1879.

Likewise, based on the lowest Akaike Information Criterion, the best-fit model for the T2D risk was the model enriched with DRI. The Akaike Information Criterion for DRI-enriched model was 1802, while the LPIR-enriched model was 1818.

Considering that DRI values are higher in men, the association was analyzed separately in men and women using different cutoff values (55 for women and 65 for men) based on the distributions of DRI in the MESA study [16] DRI distributions. The association was found to be stronger in women than in men when using these cutoff values in crude- and age-adjusted analysis. The age-adjusted HR for women was 5.65 (3.67–8.72; *p* < 0.001), and the HR for men was 3.92 (2.88–5.33; *p* < 0.001). Consistently, the HR of DRI per 1 standard deviation (SD) increment in women was higher than in men. The HR for women was 2.71 (2.29–3.22; *p* < 0.001) and 1.99 (1.72; 2.31; *p* < 0.001), for men in crude models. Furthermore, the difference between women and men persisted in age-adjusted models, HR for women was 2.50 (2.10–2.97; *p* < 0.001) and HR for men 2.04 (1.75–2.37; *p* < 0.001) (Supplemental Tables S2 and S3).

## 4. Discussion

In this large prospective cohort, comprising 6134 participants, we report for the first time that higher values of DRI, a newly developed diabetes risk algorithm, are associated with incidences of T2D. In addition, sex-stratified analyses revealed that the positive association of DRI with T2D was present in both men and women, even after adjustment for multiple T2D risk factors, including insulin resistance and BMI. Addition of DRI to the traditional predictive model improved the predictive ability for T2D. Additionally, the DRI enhanced model improved reclassification of participants across clinical risk categories for T2D compared to the Framingham Offspring Study model, and also to the previous reported model that include LP-IR, but not BCAA [19].

DRI includes the information of six lipoprotein parameters: the weighted average sizes of very low-density lipoprotein, low-density lipoprotein and HDL, along with concentrations of large very low-density lipoprotein, small low-density lipoprotein, and large HDL particles; such parameters are integrated in the LP-IR score. Although this is the first study that investigated the association between DRI and incidence of T2D, previous studies have revealed an association between LP-IR and T2D. LP-IR has been consistently found to be associated with risk of T2D in four large prospective studies, some of them with a remarkably large number of participants, i.e., the Women’s Health Study, comprising 25,925 participants free from T2D at baseline [17]. LP-IR has also been evaluated in subject of various ethnicities, i.e., the Multi-Ethnic Study of Atherosclerosis, comprising 5314 adults [16]. In addition to the lipoprotein parameters, DRI also includes information of plasma concentrations of BCAA, which have been associated with T2D risk in several recent studies [24,25,26,27,28,29].

Consistent with previous studies, we found in our study population that high values of DRI associate with increased risk of T2D. Interestingly, the highest quartile of DRI was comprised predominantly of men (79.2%), in comparison with the first quartile (16.4%). Such a difference in the proportion of the men and women among the quartiles of DRI is consistent with the reported in percentages in our previous LP-IR study, on which 72.7% of the participants in the 4th quartile of LP-IR were men and 28.6% in the 1st quartile of LP-IR were men. The increased proportion of men in the highest quartile of DRI, compared to LP-IR, could be explained by the fact that both plasma concentrations of BCAA and dietary intake of BCAA-rich foods are higher in men [45,46]. There is evidence suggesting that such differences may at least in part be attributed to differences in dietary patterns between men and women [47].

The association of BCAA with risk of T2D has been previously reported in this cohort [24]. Despite the fact that due to the design of our study we were not able to evaluate a causal mechanism, mendelian randomization studies have provided evidence about the causal relationship between BCAA and T2D [48]. Additionally, it has been described that the association of BCAA with T2D is independent of insulin resistance at baseline [29]. In the present study we demonstrated that the association of DRI with T2D was also independent of baseline insulin resistance, as assessed by HOMA-IR.

Plasma concentrations of BCAA have been found as a suitable metabolic predictor of insulin sensitivity improvement in overweight individuals after a lifestyle intervention [49]. Considering that lifestyle interventions have important health benefits, including T2D postponement [50], DRI could be a potential tool to assess the effect of lifestyle intervention in order to improve its efficacy. Future research is needed to evaluate the utility of DRI on that regard.

The present study demonstrated that DRI enhances the performance of a typical T2D predictive model based on clinical data. Clinical data-based risk scores for T2D offer several advantages, like affordability and a non-invasive nature; nevertheless, it has been reported that the probability estimates may be compromised, due several methodological flaws [51]. Furthermore, it has been reported that the performance of non-invasive risk scores varies with age, sex, BMI and country [52]. Finally, despite the fact that T2D is highly prevalent in developing countries, there is a scarcity of T2D risk scores, and the few available risk scores present several methodological limitations [53].

Besides the development of non-invasive risk scores, advances in genetics have allowed the identification of hundreds of genes associated with risk of T2D development. Although the role of singles genes in the onset of T2D is small, the combination of several genes into polygenic risk scores enhances the ability to identify patterns of disease predisposition, which could help to improve the clinical management of patients. Nevertheless, at this time, the use of genetic risk scores still has several limitations such as the fact that the current genetic scores were developed mainly in European populations, as well as the increased costs associated with genetic data acquisition and the full cost of genetic screening implementation [54]. Instead, the two main components of DRI have been demonstrated to have applicability in participants with varied ethnic backgrounds. The incremental cost of calculating DRI from data acquired for the NMR *LipoProfile Test* (which includes the calculation of LP-IR) is small, lending itself to the ability to screen patients for T2D risk. Furthermore, DRI is able to stratify a patient’s T2D risk, even when glucose levels are in the normoglycemic and prediabetic range, allowing for early identification and treatment with the goal of reducing progression to T2D.

We acknowledge several strengths of the present study. Our study included a large number of participants with a wide age range which allowed us to adjust our analysis with sufficient statistical power. Another strength of the present study is the implementation of a robust method of BCAA quantification by means of NMR. To the best of our knowledge this study explores for first time the performance of a test comprising the dual factors of lipoprotein subspecies and BCAA in the context of T2D risk assessment.

We are also aware of the limitations of the study. The PREVEND population is mainly comprised of individuals with European ancestry, which limits the generalizability of our findings to persons with different ethnicities. We did not have measurements of insulin beyond baseline assessment, which impedes us from evaluating the evolution of insulin resistance and its association with DRI. This fact limits our capacity to describe the underlying biological mechanisms. Moreover, because of the absence of repeated BCAA and LP-IR measurements, we were not able to correct for regression dilution. Finally, it should be emphasized that the validity of the LP-IR score and, as presently described the DRI score, relies on a NMR-derived laboratory parameters obtained from a single plasma specimen but does not account for genetic influences on diabetes risk.

## 5. Conclusions

In this prospective cohort study, high values of DRI, a NMR spectroscopy-measured multimarker of lipoprotein subclasses and BCAA, are associated with an increased risk of developing T2D in both men and women in the general population during extended follow-up.

## Figures and Tables

**Table 1:** Baseline characteristics by quartile of DRI scores in PREVEND participants free of T2D at baseline (*n* = 6134).

		Quartiles of DRI				
Variables	All Participants	Q1	Q2	Q3	Q4	*p*-Value
Participants, *n*	6134	1455	1596	1489	1594	
DRI score	33 (19–48)	11 (5–15)	25 (22–29)	39 (36–43)	58 (52–64)	<0.001
Sex, men, %	49.3	16.4	42.7	56.7	79.2	<0.001
Age, years	53.2 ± 12.0	50.6 ± 11.7	53.0 ± 12.5	54.5 ± 11.9	54.5 ± 11.3	<0.001
BMI, kg/m2	26.5 ± 4.2	24.2 ± 3.4	25.7 ± 3.8	27.2 ± 4.2	28.7 ± 4.0	<0.001
SBP, mm Hg	125.8 ± 18.6	118.4 ±17.8	123.1 ±18.1	127.5 ± 17.9	132.7 ± 17.5	<0.001
DBP, mm Hg	73.2 ± 9.0	69.5 ± 8.9	71.9 ± 8.7	74.3 ± 8.5	76.9 ± 8.4	<0.001
Parental history of T2DM, yes, %	14.4	11.9	13.7	15.1	16.7	<0.001
Smoking status						<0.001
Never, %	28.5	34.4	30.3	25.9	23.9	
Former, %	42.1	37.2	41.4	44.3	45.3	
Current, %	28.1	26.9	27.3	28.3	29.9	
Alcohol consumption						<0.001
<1 drinks/week, %	24.1	23.7	24.0	23.8	21.8	
1–7 drinks/week, %	48.6	50.0	51.9	46.5	46.1	
>7 drinks/week, %	26.3	21.7	23.4	28.0	31.4	
Antihypertensive drugs, %	18.2	10.3	15.9	20.1	25.8	<0.001
Lipid-lowering drugs, %	7.2	3.4	5.8	8.1	11.0	<0.001
TC, mmol/L	5.4 ± 1.0	5.2 ± 1.0	5.3 ± 1.0	5.5 ± 1.0	5.7 ± 1.0	<0.001
HDL-C, mmol/L	1.2 ± 0.3	1.5 ± 0.3	1.3 ± 0.3	1.2 ± 0.3	1.0 ± 0.2	<0.001
LDL-C, mmol/L	2.9 ± 0.7	2.7 ± 0.7	2.9 ± 0.7	3.0 ± 0.7	3.0 ± 0.8	<0.001
TG, mmol/L	1.1 (0.8–1.6)	0.8 (0.6–0.9)	0.9 (0.7–1.2)	1.2 (1.0–1.5)	1.9 (1.4–2.4)	<0.001
Glucose, mmol/L	4.8 ± 0.6	4.6 ± 0.5	4.8 ± 0.6	4.9 ± 0.6	5.0 ± 0.7	<0.001
Total BCAA, μM	377.0 ± 72.6	301.7 ± 35.1	354.9 ± 38.8	393.8 ± 44.4	455.0 ± 61.5	<0.001
Valine, μM	207.1 ± 37.2	171.0 ± 21.4	197.4 ± 24.1	216.0 ± 26.6	241.4 ± 33.7	<0.001
Leucine, μM	127.2 ± 27.7	99.1 ±13.9	118.8 ± 15.1	131.7 ± 17.2	156.5 ± 24.6	<0.001
LP-IR score	40 (21–61)	15 (8–23)	29 (20–39)	47 (38–57)	73 (62–85)	<0.001
Large VLDL-P, nmol/L	3.3 (1.6–6.6)	1.4 (0.75–2.2)	2.3 (1.4–3.7)	4.1 (2.7–6.4)	9.2 (6.1–13.8)	<0.001
VLDL size, nm	49.8 ± 9.1	45.7 ± 8.3	46.9 ± 7.5	49.6 ± 7.6	56.5 ± 8.7	<0.001
Small LDL-P, nmol/L	336 (189–534)	166 (1–274)	274 (166–289)	378 (251–522)	624 (434–848)	<0.001
LDL size, nm	20.9 ± 1.6	21.2 ± 1.8	21.1 ± 1.9	21.0 ± 1.1	20.5 ± 1.1	<0.001
Large HDL-P, μmol/L	5.1 ± 2.8	7.6 ± 4.7	5.8 ± 2.4	4.3 ± 2.3	2.8 ± 1.6	<0.001
HDL size, nm	9.1 ± 0.6	9.7 ± 0.5	9.3 ± 0.5	9.0 ± 0.5	8.7± 0.4	<0.001

Continuous variables are reported as mean ± standard deviation, median (interquartile range) and categorical variables are reported as percentage. *p* values were determined using a one-way analysis of variance for normally distributed data, Kruskal–Wallis test for skewed distributed data, and chi-square test for categorical data and represent a significant difference across the quartiles of DRI score. Abbreviations: DRI, Diabetes Risk Index; PREVEND, Prevention of Renal and Vascular End-Stage Disease; T2D, type 2 diabetes mellitus; BMI, body mass index; SBP, systolic blood pressure; DBP, diastolic blood pressure; TC, total cholesterol; HDL-C, high-density lipoprotein cholesterol; LDL-C, low-density lipoprotein cholesterol; TG, triglycerides; BCAA, branched chain amino acids; LP-IR, Lipoprotein Insulin Resistance Index; VLDL-P, very low-density lipoprotein particles; LDL-P, low-density lipoprotein particles; HDL-P, high-density lipoprotein particles.

**Table 2:** Association of DRI scores with incident T2D by quartile in the PREVEND study (*n*= 6134).

	Q1	Q2		Q3		Q4		DRI Per 1 SD Increment	
	DRI < 19	DRI 19–33		DRI 33–48		DRI > 48			
**Participants, *n***	1455	1596		1489		1594		6134	
**Events, *n***	15	39		71		181		306	
		**HR (95 % CI)**	***p*-Value**	**HR (95% CI)**	***p*-Value**	**HR (95% CI)**	***p*-Value**	**HR (95% CI)**	***p*-Value**
**Crude Model**	(ref)	2.48 (1.37; 4.50)	0.002	4.89 (2.80; 8.53)	<0.001	12.05 (7.12; 20.41)	<0.001	2.34 (2.09; 2.62)	<0.001
**Model 1**	(ref)	2.42 (1.33; 4.40)	0.003	4.69 (2.66; 8.26)	<0.001	12.07 (6.97; 20.89)	<0.001	2.46 (2.17; 2.80)	<0.001
**Model 2**	(ref)	1.84 (1.01; 3.36)	0.04	2.83 (1.59; 5.04)	<0.001	6.01 (3.42; 10.58)	<0.001	2.02 (1.76; 2.31)	<0.001
**Model 3**	(ref)	1.71 (0.93; 3.14)	0.08	2.22 (1.23; 4.03)	0.008	3.20 (1.73; 5.95)	<0.001	1.50 (1.25; 1.79)	0.001

Data are presented as HRs with 95% CIs. Model 1: Model adjusted for age and sex. Model 2: Model 1 + BMI + family history of type 2 diabetes + alcohol consumption. Model 3: Model 2 + DBP + TC+ TG + HDL-C + HOMA-IR. Abbreviations: DRI, Diabetes Risk Index; T2D, type 2 diabetes mellitus; PREVEND, Prevention of Renal and Vascular End-Stage Disease; HR, hazard ratio; CI, confidence intervals; BMI, body mass index; DBP, diastolic blood pressure; TC, total cholesterol; TG, triglycerides; HDL-C, high-density lipoprotein cholesterol; HOMA-IR, Homeostasis Model Assessment Insulin Resistance.

**Table 3:** Association of DRI scores with incident T2DM by sex-stratified quartiles in the PREVEND study (*n*= 6134).

	Q1	Q2		Q3		Q4	
	♀ DRI < 13	♀ DRI 13–23		♀ DRI 23–36		♀ DRI > 36	
	♂ DRI < 30	♂ DRI 30–43		♂ DRI 43–56		♂ DRI > 56	
**Participants, *n***	1628	1494		1534		1478	
**Males, %**	48.6	50.7		49.4		48.8	
**Events, *n***	28	42		70		166	
		**HR (95% CI)**	***p*-Value**	**HR (95% CI)**	***p*-Value**	**HR (95% CI)**	***p*-Value**
**Crude Model**	(ref)	1.63 (1.00; 2.65)	0.05	2.87 (1.84; 4.47)	<0.001	7.27 (4.84; 10.92)	<0.001
**Model 1**	(ref)	1.55 (0.95; 2.52)	0.08	2.62 (1.68; 4.09)	<0.001	6.74 (4.49; 10.13)	<0.001
**Model 2**	(ref)	1.34 (0.81; 2.20)	0.25	1.75 (1.10; 2.78)	0.018	3.75 (2.44; 5.76)	<0.001
**Model 3**	(ref)	1.30 (0.77; 2.20)	0.32	1.35 (0.81; 2.24)	0.25	1.80 (1.07; 3.02)	0.02

Data are presented as HRs with 95% CIs. Model 1: Model adjusted for age and sex. Model 2: Model 1 + BMI + family history of type 2 diabetes + alcohol consumption. Model 3: Model 2 + DBP + TC+ TG + HDL-C + HOMA-IR. Abbreviations: DRI, Diabetes Risk Index; T2DM, type 2 diabetes mellitus; PREVEND, Prevention of Renal and Vascular End-Stage Disease; HR, hazard ratio; CI, confidence intervals; BMI, body mass index; DBP, diastolic blood pressure; TC, total cholesterol; TG, triglycerides; HDL-C, high-density lipoprotein cholesterol; HOMA-IR, Homeostasis Model Assessment Insulin Resistance.

## Supplementary Materials

The following are available online at https://www.mdpi.com/2077-0383/9/9/2781/s1, Table S1: Prospective associations of DRI with risk of T2D in females, Table S2: Prospective associations of DRI with risk of T2D in males, Table S3: Prospective associations of DRI with risk of T2D in males.
