# Supplementary material for: A Newly Developed Diabetes Risk Index, Based on Lipoprotein Subfractions and Branched Chain Amino Acids, is Associated with Incident Type 2 Diabetes Mellitus in the PREVEND Cohort
Source: J Clin Med. 2020 Aug 27;9(9):2781. doi: 10.3390/jcm9092781 (PMC7563197; doi:10.3390/jcm9092781)
Supplement: Supplementary file 1 [file jcm-09-02781-s001.pdf]

Supplemental Table 1. Baseline characteristics by sex-stratified quartiles of DRI scores in PREVEND participants free of T2D at baseline (n=6134).

	Quartiles of DRI				
	**Q1**	**Q2**	**Q3**	**Q4**	**P-Value**
Participants, n	1628	1494	1534	1478	
DRI score	12 (6-22)	31 (18-37)	36 (29-50)	59 (46-65)	<0.001
Sex, men, %	48.6	50.7	49.1	48.4	0.97
Age, years	49.0 ± 12.5	51.5 ± 12.0	53.1 ± 12.1	53.8 ± 10.8	<0.001
BMI, kg/m2	24.2 ± 3.3	25.7 ± 3.4	27.2 ± 4.2	28.9 ± 4.3	<0.001
SBP, mm Hg	120.9 ±18.0	123.9 ±18.3	126.9 ± 18.4	130.9 ± 18.0	<0.001
DBP, mm Hg	70.9± 9.1	72.8 ± 9.1	73.8 ± 8.7	75.6 ± 8.6	<0.001
Parental history of T2DM, yes, %	11.2	13.6	14.9	18.2	<0.001
Smoking status					<0.001
Never, %	32.6	28.8	26.7	25.8	
Former, %	39.8	42.6	43.2	42.9	
Current, %	26.1	27.2	29.3	30.2	
Alcohol consumption					0.01
<1 drinks/week, %	23.2	23.2	23.7	26.7	
1-7 drinks/week, %	51.2	50.1	47.3	45.7	
>7 drinks/week, %	24.4	25.6	28.3	26.9	
Antihypertensive drugs, %	12.3	14.7	20.6	25.6	<0.001
Lipid-lowering drugs, %	3.6	6.5	8.8	10.0	<0.001
TC, mmol/L	5.2±1.0	5.3±1.0	5.5±1.0	5.8 ± 1.1	<0.001
HDL-C, mmol/L	1.4 ± 0.3	1.3 ± 0.3	1.8 ± 0.3	1.1 ± 0.2	<0.001
LDL-C, mmol/L	2.7 ± 0.7	2.9 ± 0.7	3.0 ± 0.8	3.1 ± 0.8	<0.001
TG, mmol/L	0.8 (0.6–0.8)	1.0 (0.8–1.2)	1.3 (1.0–1.6)	1.9 (1.4-2.5)	<0.001
Glucose, mmol/L	4.7 ± 0.6	4.8 ± 0.6	4.9 ± 0.7	5.0 ± 0.7	<0.001
Total BCAA, μM	315.2 ± 48.7	358.9 ± 50.1	389.2 ± 55.5	436.2 ± 70.2	<0.001
Valine, μM	179.2 ± 27.1	201.2 ± 28.6	215.1 ± 31.4	235.4 ± 36.7	<0.001
Leucine, μM	103.4 ±18.1	119.8 ± 18.7	131.8 ± 20.9	149.9 ± 27.1	<0.001
LP-IR score	17 (10-26)	32 (21-43)	48 (36-61)	72 (59-85)	<0.001

Continuous variables are reported as mean ± standard deviation, median (interquartile range) and categorical variables are reported as percentage. P values were determined using a one-way analysis of variance for normally distributed data, Kruskal-Wallis test for skewed distributed data, and chi-square test for categorical

data and represent a significant difference across the quartiles of DRI score.
Abbreviations: DRI, Diabetes Risk Index; PREVEND, Prevention of Renal and Vascular End-Stage Disease; T2D, type 2 diabetes mellitus; BMI, body mass index; SBP, systolic blood pressure; DBP, diastolic blood pressure; TC, total cholesterol; HDL-C, high density lipoprotein cholesterol; LDL-C, low density lipoprotein cholesterol; TG, triglycerides; BCAA, branched chain amino acids; LP-IR, Lipoprotein Insulin Resistance Index.

Supplemental Table 2. Prospective associations of DRI with risk of T2D in females.

	DRI Per 1 SD Increment		DRI <55	DRI ≥ 55	
**Participants, n**	3106		2934	172	
**Events, n**	113		84	29	
	**HR (95 % CI)**	**P-Value**		**HR (95 % CI)**	**P-Value**
**Crude Model**	2.71 [2.29;3.22]	<0.001	(ref)	6.85 [4.46;10.54]	<0.001
**Model 1**	2.50 [2.10;2.97]	<0.001	(ref)	5.65 [3.67;8.72]	<0.001
**Model 2**	2.12 [1.75;2.55]	<0.001	(ref)	3.82 [2.45;5.96]	<0.001
**Model 3**	1.49 [1.12;1.98]	0.006	(ref)	1.16 [0.63;2.13]	0.63

Data are presented as hazard ratios (HRs) with 95% confidence intervals (CIs).
Model 1: Model adjusted for age.
Model 2: Model 1 + BMI + family history of type 2 diabetes + alcohol consumption.
Model 3: Model 2 + DBP + TC+ TG + HDL-C + HOMA-IR.

Abbreviations: DRI, Diabetes Risk Index; T2DM, type 2 diabetes mellitus; HR, hazard ratio; CI, confidence intervals; BMI, body mass index; DBP, diastolic blood pressure; TC, total cholesterol; TG, triglycerides; HDL-C, high density lipoprotein cholesterol; HOMA-IR, Homeostasis Model Assessment Insulin Resistance.

Supplemental Table 3. Prospective associations of DRI with risk of T2D in males.

	DRI Per 1 SD Increment		DRI <65	DRI >= 65	
**Participants, n**	3028		2684	344	
**Events, n**	193		134	59	
	**HR (95 % CI)**	**P-Value**		**HR (95 % CI)**	**P-Value**
**Crude Model**	1.99 [1.72;2.31]	<0.001	(ref)	3.73 [2.75;5.07]	<0.001
**Model 1**	2.04 [1.75;2.37]	<0.001	(ref)	3.92 [2.88;5.33]	<0.001
**Model 2**	1.59 [1.34;1.88]	<0.001	(ref)	2.45 [1.76;3.42]	<0.001
**Model 3**	1.19 [0.96;1.48]	0.11	(ref)	1.36 [0.91;2.03]	0.14

Data are presented as hazard ratios (HRs) with 95% confidence intervals (CIs).
Model 1: Model adjusted for age.
Model 2: Model 1 + BMI + family history of type 2 diabetes + alcohol consumption.
Model 3: Model 2 + DBP + TC+ TG + HDL-C + HOMA-IR.

Abbreviations: DRI, Diabetes Risk Index; T2DM, type 2 diabetes mellitus; HR, hazard ratio; CI, confidence intervals; BMI, body mass index; DBP, diastolic blood pressure; TC, total cholesterol; TG, triglycerides; HDL-C, high density lipoprotein cholesterol; HOMA-IR, Homeostasis Model Assessment Insulin Resistance.
